# The Influence of Wildfires on Aerosol Size Distributions in Rural Areas

**DOI:** 10.1100/2012/735697

**Published:** 2012-05-02

**Authors:** E. Alonso-Blanco, A. I. Calvo, R. Fraile, A. Castro

**Affiliations:** ^1^Department of Physics (IMARENAB), University of León, León 24071, Spain; ^2^Centre for Environmental and Marine Studies (CESAM), University of Aveiro, Aveiro 3810-193, Portugal

## Abstract

The number of particles and their size distributions were measured in a rural area, during the summer, using a PCASP-X. The aim was to study the influence of wildfires on particle size distributions. The comparative studies carried out reveal an average increase of around ten times in the number of particles in the fine mode, especially in sizes between 0.10 and 0.14 **μ**m, where the increase is of nearly 20 times. An analysis carried out at three different points in time—before, during, and after the passing of the smoke plume from the wildfires—shows that the mean geometric diameter of the fine mode in the measurements affected by the fire is smaller than the one obtained in the measurements carried out immediately before and after (0.14 **μ**m) and presents average values of 0.11 **μ**m.

## 1. Introduction

The size of atmospheric aerosols depends greatly on the sources and sinks, as well as on the meteorological processes dominating during their lifetime [1–4]. Biomass burning is one of the most important contributors of aerosols to the atmosphere, releasing large amounts of particles and gases, and causing alterations in atmospheric composition at a local and at a global scale. The effects aerosols may trigger in the atmosphere depend on the type of material burnt, the combustion phase, the relative humidity, and the wind conditions [5]. A growing number of studies focus on these changes, because aerosols undergo complex interactions in the atmosphere and condition global energy balance [6].

Many studies have found an increased number of aerosols in the fine or accumulation mode during wildfires [7–9]. An in-depth analysis of the emissions released by biomass burning can be found in Reid et al. [5]. This study showed that out of all the particles released during these phenomena, between 80% and 90% correspond to this mode, with a count median diameter (CMD) of 0.13 *μ*m. These particles are mainly composed of organic material; inorganic elements only account for approximately 10% of the mass [7].

The water-absorption capacity of these particles varies greatly. The hygroscopicity of aerosols originated by biomass burning depends on the internal composition of the organic and inorganic material of the submicrometer particles [10–12]. In consequence, studies on aerosol size distributions must take into account hygroscopicity. The ability of aerosols to absorb water vapor from the atmosphere is an important feature, but very difficult to study [13]. The water content in the particles affects factors such as size, total mass, acidity, the amount of water-soluble substances they contain, light-dispersion properties, chemical reactivity, the ability to function as condensation nuclei in a cloud, and their permanence in the atmosphere.

The ability of aerosols generated by biomass burning to form condensation nuclei has been the focus of several studies. Authors such as Warner and Twomey [14], Eagan et al. [15], and Roberts et al. [16] have found that, at supersaturations higher than 0.5%, the particles generated by wildfires function as condensation nuclei (CCN). More recently, Petters et al. [17] studied the hygroscopic properties of aerosols freshly emitted from laboratory biomass burning experiments. They conclude that at the point of emission, most particles are CCN active and do not depend upon conversion in the atmosphere to more hygroscopic compositions before they can participate in cloud formation and undergo wet deposition.

In this paper, we will analyze the characteristics of particle size distributions in a rural area in the summer months, and the changes in these distributions caused by the arrival of aerosols from biomass burning, mainly wildfires affecting no-tree woodland, but sometimes also trees, and crops or crop stubble. These wildfires are particularly frequent in the rural study zone. The wildfires considered are all the fires registered in the province of León, Spain. The distance with the probe installed at the sampling site varies between a few kilometres and a maximum of 70 km.

In the case of wildfires, the source of the particles is well defined and located; but in the study of aerosol size distributions, we must also take into account the origin and the type of air mass that carries the particulate matter when it reaches the sampling site [18].

## 2. Study Zone

**Figure 1:**
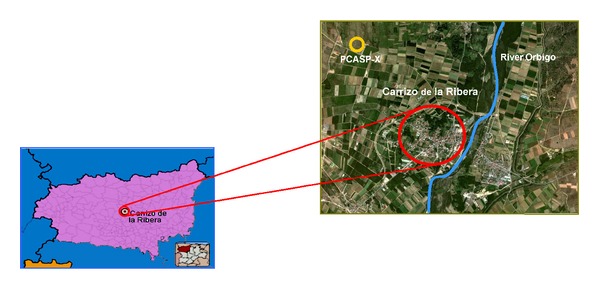
Location of Carrizo de la Ribera in the province of León. Location of the PCASP-X probe and the river Órbigo, with respect to the town.

The measurements have been carried out in the district of Carrizo de la Ribera, in the middle of the province of León, Spain, north-west of the capital (Figure 1). This rural area lies at 873 m above sea level and has 2,554 inhabitants. Farming is the main economic activity, and hop the most widely grown crop. The weather is monitored by a station from the Spanish National Weather Agency installed in that town (42°35′N, 5°39′W).

The study zone lies on the left bank of the River Órbigo, over a fluvial terrace. The climate is of the continentalized Mediterranean type, with a marked seasonality. Precipitation is scattered irregularly along the year, with minimum values in the summer and the highest values in spring and autumn.

Temperatures are fresh, with an annual average of 10.5°C. The winters are cold, with frequent frosts. The summers are warm, with maximum temperatures that may be over 35°C, although nocturnal temperatures are moderate, as it is an area of irrigated farming systems where land is mainly watered by flooding.

## 3. Materials and Methods

### 3.1. Measurement Equipment

A laser spectrometer (Passive Cavity Aerosol Spectrometer Probe, PMS Model PCASP-X) was installed in a field 2 km from Carrizo de la Ribera (42°35′59′′ N, 5°50′50′′ W) to determine aerosol size spectra in the rural areas. This device measures particles ranging between 0.1 and 10 *μ*m considering their light-dispersion properties in a wave length of 633 nm between the angles of 35° and 135°. The spectrometer measures 31 channels, that is, 31 discrete particle size intervals. The probe was calibrated by the manufacturer using polystyrene latex particles of a known size. The refractive index of latex beads (1.59–0 i) is different from that of atmospheric particles, resulting in an aerosol size distribution that is “latex equivalent”.

Here, we are presenting PCASP-X size distributions corrected using Mie theory and implemented with a computer code developed by Bohren and Huffman [19], according to the refractive index typical of rural aerosols, whose value varies depending on the relative humidity [20]. The refractive indices were calculated interpolating the real part and the imaginary part in accordance with the relative humidity at the time of measurement (values between 17% and 100%).  Table 1 shows the refraction indices used for the interpolation.

It was also necessary to carry out a number of corrections on the number of counts sampled by the spectrometer in each channel. First, the flow measurement value was set in relation to the altitude of the sampling point; the probe was installed at 834 m introducing a correction factor of 0.905. In addition, each measurement was corrected according to the activity registered. Finally, the last correction was determined by the duration of the samplings, which was 600 seconds. These corrections are described in more detail in Calvo et al. [6].

The study period covers the 122 days of the months of June, July, August, and September of the year 2000. Eight measurements were carried out daily, measuring the ambient particle size spectrum in the rural study area automatically, during 15-minute intervals every 3 hours.

The probe was installed next to a weather station registering automatically data on precipitation, pressure, temperature, relative humidity, and wind speed and direction. A wind profiler was also used (Sodar SR1000), with a pulse frequency of 5 tones, around 2.150 Hz, pulse power of 300 W, a pulse-repetition period of 8 s, and a maximum range of 1.250 m. This device registered automatically data from the three wind components. The data registered by the weather station and the data registered by the Sodar were all stored on a computer every 30 minutes.

### 3.2. Thermal Inversions and Circulation Weather Type Classification

The thermal inversions have been calculated using data from soundings in La Coruña (43.36°N, 8.41°W, altitude 67 m), Madrid (40.50°N, 3.58°W, altitude 633 m), and Santander (43.48°N, 3.80°W, altitude 59 m) provided by the University of Wyoming (http://weather.uwyo.edu/upperair/sounding.html).

In order to identify the type of weather associated to a particular synoptic situation, a Circulation Weather Type classification (CWTs) was designed based on Jenkinson and Collison [21] and Jones et al. [22]. These procedures were initially developed to define objectively Lamb Weather Types [23] for the British Isles. The daily circulation affecting the Iberian Peninsula is described using a set of Indices associated to the direction and vorticity of the geostrophic flow. The Indices used were the following: southerly flow (SF), westerly flow (WF), total flow (F), southerly shear vorticity (ZS), westerly shear vorticity (ZW), and total shear vorticity (Z). These Indices were computed using sea level pressure (SLP) values obtained for the 16 grid points, distributed around the Iberian Peninsula. This method allows for a maximum of 26 different CWTs. Following Trigo and DaCamara [24] in their study for Portugal, this study does not have an unclassified class, but rather opts for disseminating the fairly few cases (<2%) with possible unclassified situations among the retained classes. This classification has already been used on the Iberian Peninsula, for example, in the study of lightning [25], splash erosion [26], or aerosol size distribution in precipitation events [27].

### 3.3. Data Analysis

**Figure 2:**
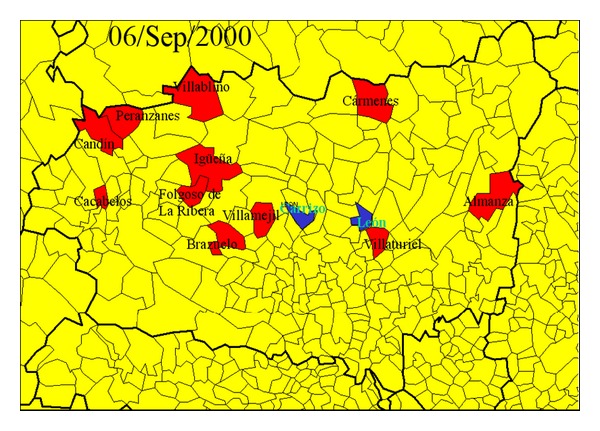
Example of a map representing the districts in the province of León, Spain, with active wildfires on 6th September 2000. The probe was installed in Carrizo.

The Department for the Environment of the regional government of the Junta of Castile and León provided the database with the number of wildfires, the district where each fire occurred, the date of detection and extinction (with exact date and time), the land area affected in hectares (ha), and the type of vegetation burnt during the summer months (June, July, August, and September). This information was used to draw for each day a map of the province of León highlighting all the districts affected by wildfires. One of these maps is shown in Figure 2.

This material and the data on wind direction at surface level and at a certain altitude, registered by the weather station and the Sodar, were used to identify the measurements carried out by the aerosol probe that could have been affected by the transport of the smoke plumes from any nearby wildfires. The changes in the number of particles revealed the arrival of a plume at the sampling site. In general, a plume affected only 1 out of the 8 measurements carried out daily, as these measurements were carried out every 3 hours. However, in the case of large fires several measurements may be affected in one day or even over several subsequent days, depending on the duration of the fire and the intensity of the wind.

The particle measurements of the 8 daily registers were compared with the time interval of the wildfire, from detection to extinction. The measurements with very large numbers of particles were thus identified. In some cases, there were 20-fold increases with respect to the other measurements registered the same day. After identifying the measurements that were possibly affected by the arrival of a smoke plume to the probe, the data were confirmed with an analysis of the wind direction and speed and the distance between the wildfire and the probe. The increases in the measurements that could not be explained by these variables were excluded from the study.

It must be taken into account that on some days high levels of particles may be explained by the arrival of smoke plumes from wildfires in other provinces close to the province of León, especially Zamora and Orense. However, the data on the wildfires in those provinces during the study period were not made available.

Two different databases were built using the information obtained: one includes the measurements affected by the smoke plumes carried by the wind towards the probe (41 measurements in 28 different days out of the 122 study days); the other one comprises the measurements that were not affected by the smoke plumes from surrounding fires (935 measurements in 121 different days out of the 122 study days). Eight measurements were carried out every single day, so there are both affected and non-affected measurements on most days.

The aerosol size distribution and the daily CWT were analyzed, considering the affected measurements and the non-affected measurements separately.

In the measurements that were not affected by the wildfires the study focuses on the influence of the weather types on the number of particles and the count median diameter of the fine mode. Soundings provided data on thermal inversions, mainly radiative inversions, on the days where the number of particles was higher than average.

The distributions of the measurements affected by wildfires were studied in detail. Measurements contaminated by the fires were compared with those not contaminated, and a detailed analysis was carried out of the geometric diameter of the fine mode and the number of particles in this size range during the 8 daily registers. The evolution of the accumulation mode and of the coarse mode has been analyzed too, as well as the relative humidity at three different stages: in the measurements before the ones affected by the plumes from the fires, in the ones affected by the fires, and in the ones after the fires.

## 4. Results and Discussion

### 4.1. Meteorological Study and Circulation Weather Types

Table 2 shows the meteorological features of the months of June, July, August, and September 2000. The average monthly temperatures are typical of the summer months. The highest value is reached in July with 17.1°C. The maximum temperatures are high in all four months, over 30°C, and the minimum temperatures are around 2°C.

The monthly precipitation accumulated in the study period is low. The highest value was reached in September with 26.8 mm. The average relative humidity is around 65%, with 71% in September. The average wind speed was low, with a monthly average under 2 m/s. The high relative humidity registered, mainly during the night, are due to the irrigation system used in the fields close to the town and also to the fact that the River Órbigo flows close by at a distance of about 2.5 km.

**Figure 3:**
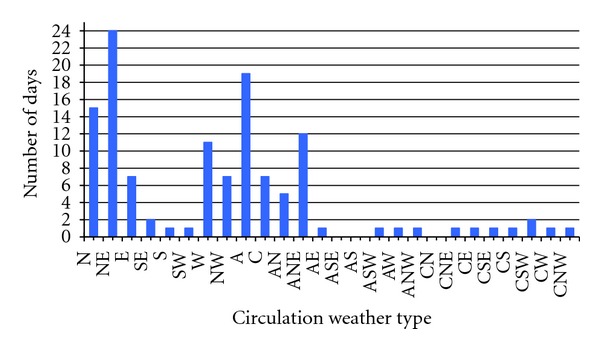
Circulation Weather Type classification in the months of June, July, August, and September 2000.

The CWT classification shows that during the months from June to September (Figure 3), the dominant CWTs are the following three: the purely north-eastern type (NE), the anti-cyclonic type (A) controlled by the geostrophic vorticity, and the purely northern type (N), with 24, 19, and 15 days, respectively. The purely directional weather types are also relatively frequent: the Eastern type (E), the Western type (W), and the Northwestern type (NW), two hybrid types with northern (AN) and northeastern direction (ANE), and one nondirectional type, the cyclonic type (C). During the summer of the year 2000, the anticyclonic weather type and the flows with a northern component were dominant over the Iberian Peninsula, following the same trend as the one found in other studies on weather types and precipitation in the whole province of León [28].

### 4.2. Characterization of the Wildfires

The province of León is the largest in the region of Castile and León, Spain, with an area of 15,581 km^2^. The climate is of the Mediterranean type with continental influence and with some areas affected by the Atlantic Ocean too. The result is a wide range of different landscapes, making León one of the provinces in Spain that suffers the highest number of wildfires with the largest areas burnt.

Most of these wildfires are deliberate. According to statistics provided by the Junta of Castile and León, 90% of wildfires are caused by humans, either deliberately or as the result of negligence.

In the summer of the year 2000, a total of 465 wildfires were registered, an average of four per day. The fires are mostly detected during the central hours of the day, between 1000 UTC and 1800 UTC, and only very few fires start during the night.

Most wildfires were classified as of medium size, burning between 1 and 500 ha. In general, it was found that the wildfires that scorch more than 30 ha last longer than the day when they were detected, and some may even be active over several days, in the case of very large fires (those affecting more than 500 ha).

During the study period, it was observed that the number of wildfires increased gradually: August was the month with the highest number of fires, 201, followed by September, with 171, whereas June and July had less than 70 fires each (Table 3). The lowest number was registered in June, with only 26 wildfires.

The total balance of hectares burnt was 20,636, affecting forest areas, no-tree woodland, and crops and crop stubble, with 87% of the land area corresponding to low-vegetation areas. The highest number of hectares burnt was registered in August and September, with over 9,500 ha per month.

The surface area affected (S) varies greatly during the summer. In June and July, it is mostly outbreaks of wildfires that are detected (S < 1 ha) or medium-size fires (500 ha > S > 1 ha), with no large fires at all (S > 500 ha). In contrast, in the months of August and September most of the fires are medium-sized with nearly 500 ha burnt, and there are 9 large fires, 5 in August and 4 in September, which together account for 45% of the total area burnt in the summer of the year 2000. The two largest fires took place on the 18th of August 2000 in the districts of Encinedo and Truchas (about 70 km from the study zone), which together burnt nearly 3,000 ha (Table 4).

The smoke plumes of these wildfires, medium and large, may be carried away by the wind to far off places, at least at a regional scale, and in this study they are responsible for the huge increases in the number of aerosols registered by the probe in the rural study zone.

### 4.3. Analysis of the Measurements “Not Contaminated” by Wildfires

#### 4.3.1. Analysis of the Weather Types

**Figure 4:**
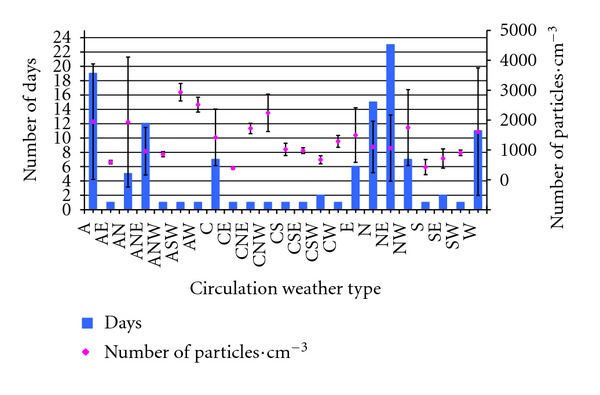
Number of particles registered and standard deviation for each Circulation Weather Type in 121 days in the months of June, July, August and September, in accordance with the days with each particular weather type.

In order to study the influence of the weather types onto the number of aerosols in the study zone, we have analyzed the measurements that were not contaminated by aerosols from the wildfires. For each weather type, we have studied the average number of total particles and the standard deviation, as well as the number of days with those weather types (Figure 4). 23% of the study days (27 out of 121) present an average content of particles cm^−3^ between 2,000 and 3,000, and the weather types registered were the anticyclonic type (A), northern anticyclonic (AN), western anti-cyclonic (AW), southwestern anti-cyclonic (ASW), and the northwestern cyclonic type (CNW). In other words, high pressures seem to favor higher numbers of aerosols, with the arrival of maritime air masses. However, the standard deviation observed on the number of particles varies greatly in the anti-cyclonic type (A) and the northern hybrid (AN). This means that with these weather types the number of particles depends greatly on the air mass. On the other hand, the following weather types have little influence in the number of aerosols, as they register mean values of less than 1,000 particles cm^−3^: eastern anti-cyclonic (AE); northeastern and northwestern anti-cyclonic (ANE & ANW), eastern, south-eastern and south-western cyclonic types (CE, CSE and CSW); the purely directional southern, south-eastern and south-western types (S, SE and SW). The standard deviations are also very low, except in the north-eastern anti-cyclonic weather type (ANE). These weather types account for only 18% of the days. However, they favor the arrival of continental air masses to the Iberian Peninsula, especially air masses from the north of Africa. In the remaining weather types, the average values lie between 1,000 and 2,000 particles cm^−3^.

Next, a comparative analysis was carried out on the relationship between the count median diameter of the fine mode (CMD_f_) and the weather types (Table 5). Four size ranges have been defined to enable us to interpret the results more easily. The particles with diameters smaller than 0.13 *μ*m are mainly detected in cyclonic weather types with southern and western wind components. The air masses that reach the Iberian Peninsula in these situations come from the north of Africa (Saharan air masses) or from the Atlantic Ocean, carrying with them marine aerosols. The particles with CMD_f_ sizes between 0.13 and 0.14 *μ*m show dominant northern and western components, although anti-cyclonic situations may generate particles in this size range too. These weather types are clearly influenced by maritime and continental air masses from Western Europe. In the case of diameters between 0.14 and 0.15 *μ*m, the weather types found are the north-eastern anti-cyclonic type and the northwestern cyclonic type, that is, the air masses that arrive at the Iberian Peninsula are both maritime and continental air masses from Eastern Europe. The largest diameters (>0.15 *μ*m) correspond to continental air masses (CNE) and the Eastern anti-cyclonic weather type (AE) with maritime aerosols from the Mediterranean.

To sum up, the air masses from the north of Africa carry smaller particles (smaller than 0.13 *μ*m) than the air masses brought to the Iberian Peninsula from continental Europe (larger than 0.13 *μ*m). With the weather types typical of maritime air masses, the sizes registered show that, in general, the aerosols are smaller than 0.14 *μ*m. In other words, even though continental air masses contribute fewer particles than maritime air masses, the aerosols they carry are larger. The arrival of European air masses has been studied by authors such as Alonso et al. [29], Gangoiti et al. [30], Viana et al. [31], or, more recently, by Escudero et al. [32].

#### 4.3.2. Temporal Evolution of the Number of Particles

**Figure 5:**
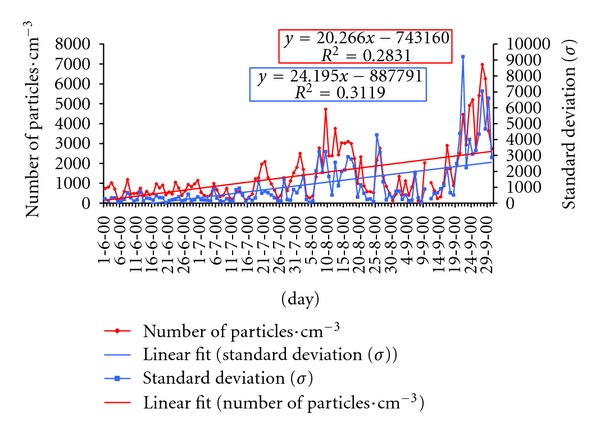
Average number of particles and standard deviation in the months of June, July, August, and September 2000.

Figure 5 shows the daily evolution of the number of particles and the standard deviation during the whole study period. Positive linear correlations were found, thus revealing that the number of particles in the study zone increases gradually over time as the summer goes by. Both Pearson correlations are significant for a significance level of 0.05. In general, the average number of particles registered daily is less than 2000 particles cm^−3^, with big differences in the measurements. There were two periods at the end of the summer with oscillations between 2000 and 7000 particles cm^−3^ (second half of August and second half of September). This increase may be due to the high number of wildfires in August and September (some days have been recorded up to 50,000 particles cm^−3^), which account for most of the land area burnt that summer in the province of León.

It must not be forgotten that the province of León is surrounded by two other provinces which are often affected by wildfires too, Orense, in the region of Galicia, to the north-west of León (the distance between both capitals is 274 km), and Zamora, in the region of Castile and León, to the south of León (the distance between the two capitals is 133 km). Both provinces are important emitters of particulate matter to the atmosphere, contributing to the increase in the number of aerosols and to changes in their size distributions.

During the 27 days comprised by these two periods (second half of August and second half of September) there were frequent thermal inversions at altitudes of less than 1000 meters (AGL), both radiative and subsidence inversions. Both types of inversion often occurred the same day (Table 6). These inversions hinder the vertical dispersion of aerosols, so their number remains high in the lower layers of the atmosphere. The days with more than 2000 particles cm^−3^ correspond mainly to the anticyclonic weather type (A), registered in six study days, followed by the cyclonic type (C), the purely directional northern (N), and north-eastern types (NE), with three days each. Therefore, the increase in the daily numbers of particles in the months of August and September should not be attributed only to the large number of wildfires raging those days, but also to the clear influence of the anti-cyclonic weather type, which favours a situation of great atmospheric stability enhancing the formation of thermal inversions.

### 4.4. Influence of Wildfires on the Measurements of Aerosol Size Distributions

The multi-log-normal function has been used to characterize the size distributions of aerosol particles [33, 34]. This enables a simple comparison between several data sets of aerosol particles to be carried out. The multi-log-normal concept is thoroughly described in the literature and the overall outcome has proven to be useful whenever parameterizations are required [35, 36]. The particle size distribution is assumed to consist of several lognormal modes:

(1) $$\begin{array}{cc} & \frac{dN\left(D\right)}{d\left(\log\left(D_{p}\right)\right)}\\ & \;=\overset{n}{\underset{i=1}{\sum }}\frac{N_{i}}{\sqrt{2\Pi }\log\left(\sigma_{g,i}\right)}\exp\left[-\frac{{\left(\log\left(D_{p}\right)-\log\left({\bar{D}}_{pg,i}\right)\right)}^{2}}{2{\left(\log\sigma_{i}\right)}^{2}}\right]. \end{array}$$

The three parameters that characterize an individual mode *i* are the mode number concentration *N*_*i*_ (cm^−3^), the mode geometric variance *σ*_*g*,*i*_^2^ (dimensionless), and the mode geometric mean diameter ${\bar{D}}_{pg,i}$ (*μ*m). *D*_*p*_ is the particle diameter and *n* is the number of individual modes. The least-square method was used to estimate the lognormal parameters *N*_*i*_, *σ*_*g*;*i*_, and ${\bar{D}}_{pg,i}$ [37]. 

For the count distribution, the geometric mean diameter, *D*_*g*_, is customarily replaced by the count median diameter or CMD. The geometric mean is the arithmetic mean of the distribution of log *D*_*p*_, which is a symmetrical normal distribution, and hence, its mean and median are equal. The median of the distribution of log *D*_*p*_ is also the median of the distribution of *D*_*p*_, as the order of values does not change in converting to logarithms. Thus, for a lognormal count distribution, *D*_*g*_ = CMD.

The size distributions analyzed using the PCASP-X were found to be bimodal, that is, they have a fine and a coarse mode. The daily number of particles in the coarse mode was very low, around 5 particles cm^−3^, so only data for the fine mode are considered in the results.

The influence of wildfires on the particle size distributions was studied taking two separate samples: on the one hand, the measurements not affected by the fires, taken before and after the plume crossed the study zone; and on the other hand, a sample of measurements affected by the smoke plumes from wildfires in a radius of 70 km around the probe. Comparative analyses were then carried out using these data to identify changes in atmospheric aerosols and their evolution during these events. 

**Figure 6:**
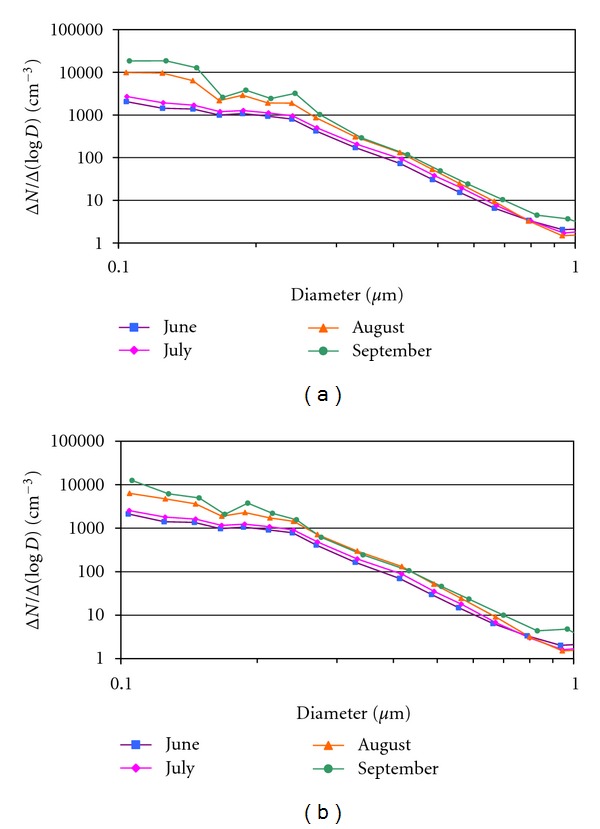
Mean size distributions in the months of June, July, August, and September 2000 in (a) the measurements registered with wildfires and in (b) measurements not contaminated by wildfires. Only the sizes between 0.1 and 1 *μ*m are shown.

The comparative analysis between the monthly aerosol size distributions including all measurements and including only data not contaminated by wildfires (Figure 6) reveals a clear increase in the number of particles between 0.1 and 0.2 *μ*m. This increase is not noticeable in the months of June and July because few wildfires are registered—26 fires in June and 67 in July, with 500 and 728 ha burnt, respectively. These fires contaminated only 6 measurements (4 in June and 2 in July) out of the 41 total measurements contaminated by particulate matter from smoke plumes between June and September. In contrast, in the months of August and September the increase in the number of particles is very sharp. We claim that this is due to the higher number of wildfires registered, 201 in August and 171 in September, burning an area of 19,428 ha distributed equally between the two months. The smoke plumes contaminated 35 measurements of the probe, 16 in August, and 19 in September, out of the 41 total.

**Figure 7:**
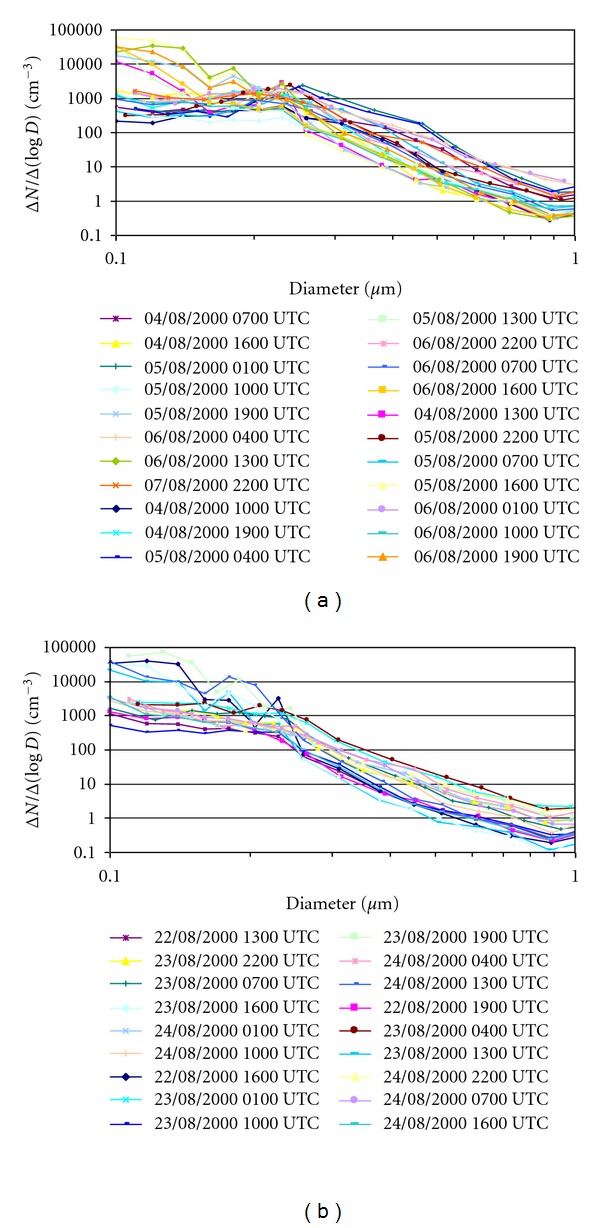
Aerosol size distributions of two time intervals: (a) between 4th August 2000 at 0700 UTC and 7th August 2000 at 2200 UTC and (b) between 22nd August 2000 at 1300 UTC and 24th August 2000 at 1600 UTC, representing measurements contaminated by wildfires. Only the sizes between 0.1 and 1 *μ*m are shown.

The increase in this size range (0.1 to 0.2 *μ*m) is observed in all the measurements contaminated by smoke in the study zone.  Figure 7 illustrates two examples: (a) between 4th August 2000 at 0700 UTC and 7th August 2000 at 2200 UTC and (b) between 22nd of August 2000 at 1300 UTC and 24th August 2000 at 1600 UTC, with 22 and 18 size distribution measurements, respectively. Both examples include measurements prior to the arrival of the smoke plume, the contaminated measurements, and the ones carried out after the smoke plume, showing a clear increase in the number of particles smaller than 0.2 *μ*m in the contaminated measurements.

**Figure 8:**
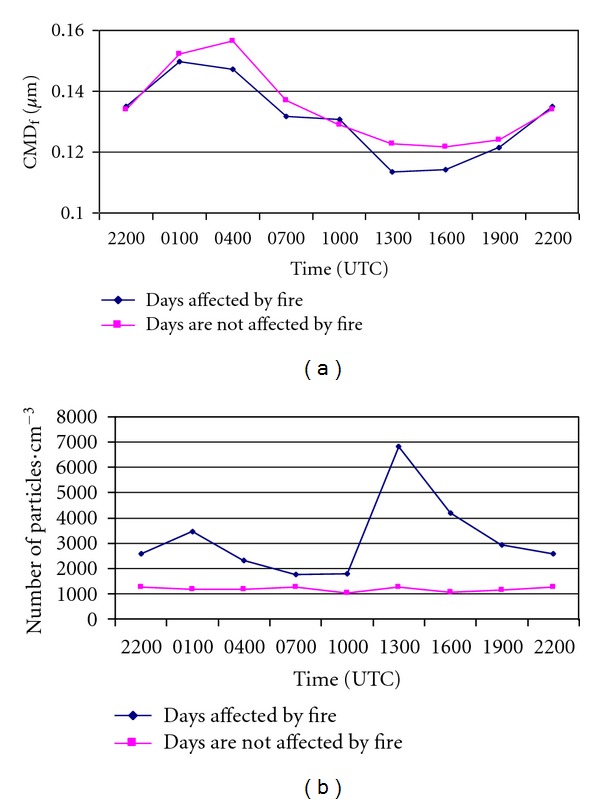
Comparative analysis of the evolution of (a) the geometric mean diameter (CMD_f_) and (b) the total number of particles in the fine mode in the 8 daily registers, for data including contaminated and noncontaminated measurements.

To determine the influence of the wildfires on the 8 daily particle measurements, we carried out a comparative analysis of the evolution of the geometric mean diameter of the fine mode (CMD_f_) and the total number of particles in affected and nonaffected measurements (Figure 8). Contaminated and noncontaminated data follow the same trend along the 8 daily measurements: there is a clear difference between the measurements taken during the night (from 1900 UTC, beginning of dusk, to 0700 UTC, beginning of dawn) and the ones taken during daylight (from 0700 UTC to 1900 UTC). In both cases, the geometric mean diameter registered during the night tends to increase until 0.15 *μ*m in contaminated measurements, and until 0.16 *μ*m in noncontaminated measurements, around 0400 UTC. From that time on, there is a decrease in the geometric mean diameter of the fine mode in both data sets, until 0.11 *μ*m in data contaminated by fires, and 0.12 *μ*m in non-contaminated data, at around 1300 UTC. This situation remains stable until 1900 UTC, when the diameter increases again. The results show that on days with wildfires the particle sizes are smaller than on days with no wildfires, both during the day and during the night.

As for the number of particles in the fine mode, we find two completely different situations when comparing affected and non-affected measurements. The data from contaminated measurements show that during the night and in the early hours (from 2200 UTC to 1000 UTC) the number of particles registered is between 2000 and 3500 particles cm^−3^. From that time on, the number of particles increases, reaching its maximum at 1300 UTC, with nearly 7,000 particles cm^−3^, coinciding with the hours registering the lowest value in the geometric mean diameter of the fine mode and with the time when the fires are most active. Initial values are back by 1900 UTC. On the other hand, in the data not contaminated by aerosols from wildfires, the number of particles in the fine mode remains stable during the whole day, around 1000 particles cm^−3^. In conclusion, the increase in the number of aerosols in the smallest fraction of the fine mode is clearly due to the contamination by particulate matter from wildfires, because there is no other anthropogenic source of aerosols nearby, as we are in a rural area.

**Figure 9:**
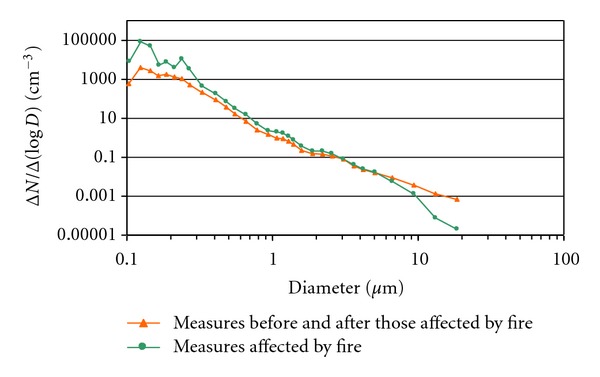
Mean size distributions of the measurements carried out immediately before and after those affected by wildfires and of the affected measurements during the months of June, July, August, and September 2000.

The aerosol size distributions in the 41 measurements contaminated by particulate matter from the wildfires were compared with the distributions in the 82 measurements taken immediately before and after the ones affected (Figure 9). Contaminated measurements had an average number of 17,000 particles cm^−3^ in the fine mode, compared to only 1,500 particles cm^−3^ on average in non-contaminated measurements. This represents an average increase in the number of particles in the fine mode of 1000%, with values of nearly 2000% in diameter ranges between 0.10 and 0.14 *μ*m.

The concentration of PM10 particulate matter was estimated considering 1.35 g cm^−3^ as the density of particles from biomass burning [38] and 1 g cm^−3^ for ambient air in rural environments. The average concentrations in measurements not contaminated by wildfires are around 14 *μ*g m^−3^, compared to an average of 86 *μ*g m^−3^ in those measurements contaminated by aerosols from wildfires. The pollution caused by wildfires exceeds the threshold value of 50 *μ*m m^−3^ established by the Spanish and European Regulation on Air Quality (Royal Decree 1073/2002 of 18th October). These remarkable increases cannot be explained by high pressure systems with atmospheric stability lasting several days, nor by the simultaneous presence of radiative and subsidence inversions. The main cause is the arrival of smoke plumes from wildfires in a radius of 70 km from the probe.

The number of particles in the fine mode increases considerably, and to know exactly what happens to the size of these particles when the probe is measuring aerosols from the wildfires, we compared the mean CMD of the fine or accumulation mode before, during, and after the smoke plumes. The influence of humidity on the growth of these particles was also analyzed (Table 7). When compared with the measurement prior to the arrival of the smoke plume, in the affected measurement, there is a clear decrease in the size of particles of around 19%, dropping from average values of CMD_f_ of 0.14 *μ*m to 0.11 *μ*m. This occurs as the number of particles with these sizes increases with the arrival of the smoke plume.

It was observed that when the relative humidity in the contaminated measurement is higher than the one in the previous measurement, a humidity of around 80%, the geometric mean diameter of the fine mode increases from 0.10 *μ*m to 0.14 *μ*m. In these cases, we claim that the aerosols derived from biomass burning undergo hygroscopic growth absorbing atmospheric water vapor, thus increasing their size. In 1959 Orr et al. [39] already found that when the relative humidity (RH) rises above 40%, even weakly soluble aerosol particles can absorb water from the air. As a consequence, this additional water increases the particle size. Once the measurements are not affected by the smoke plume from the wildfires, the CMD tends to stabilize returning to the values prior to the arrival of the plume from the fire.

## 5. Conclusions

In the summer season, the analyzed air masses from the north of Africa contributed smaller particles (smaller than 0.13 *μ*m) than the ones brought by air masses from continental Europe (larger than 0.13 *μ*m). With weather types including maritime air masses, the size ranges registered reveal that in general the aerosols are smaller than 0.14 *μ*m. That is continental air masses contribute fewer particles than maritime air masses, but these particles are larger.

The average number of particles (excluding the ones generated by biomass burning) registered daily during the study period varies between 2000 and 7000 particles cm^−3^. However, on certain days as much as 50,000 particles cm^−3^ were detected in a rural area, like the study zone, with a total lack of anthropogenic sources of pollution this implies that the cause must be the high number of wildfires registered every summer. Part of this increase may be attributed to the stable atmospheric situations typical of the summer season, as well as to the occurrence of deep radiative and/or subsidence thermal inversions. However, in this study, because of the sudden increases observed in the number of particles, the most plausible explanation is the transport of smoke plumes at a regional scale full of particulate matter.

In the province of León, wildfires burnt 20,636 ha between June and September 2000. We claim that wildfires and other large fires in the surrounding provinces are contaminating the air in rural areas during the summer, reaching average values of PM10 concentrations of 86 *μ*m·m^−3^ clearly exceeding the threshold value of 50 *μ*m·m^−3^ established by the Spanish and European Regulation on Air Quality.

The wildfires cause not only a considerable increase in the number of particles in the atmosphere but also changes in particle size distributions. Average increases of around 1000% have been found in the number of particles in the fine mode. In particles between 0.10 and 0.14 *μ*m, the increases reach 2000%, revealing that wildfires clearly contribute particles with these sizes. The air affected by the fires presents aerosols with average values of the CMD of the fine mode around 0.11 *μ*m versus average values of 0.14 *μ*m in clean rural air.

When the relative humidity increases in the measurements contaminated by the fires when compared with the non-contaminated ones (RH around 80%), the CMD of the fine mode increases by 50%. This may be due to the hygroscopic growth of the aerosol, which absorbs water vapor from ambient air, from the river Órbigo, and from the irrigation system in the study zone.

Summing up, in a rural area, with few anthropogenic sources of aerosols, the considerable increases registered in the number of particles cannot be explained by high pressures leading to atmospheric stability, nor by thermal inversions, but must be attributed to wildfires, large or small, with smoke plumes covering sometimes great distances and causing important ambient pollution of nonanthropogenic origin.

The authors believe this research line must be pursued because the devastating consequences of wildfires cause immediate changes in atmospheric composition at local and regional scales, and the increased number of aerosols produce changes in the Earth's global radiation balance, causing both direct and indirect radiative forcing. Moreover, exposure to atmospheric particulate matter may have adverse effects on human health.

## Figures and Tables

**Table 1:** Refractive Index of Atmospheric Aerosol Particle at a He-Ne Laser Wavelength (0.6328 *μ*m) for Rural Aerosol Model (according to Kim and Boatman, [20]).

Relative humidity (%)	Rural aerosol
0	1.530–6.60 × 10^−3^ *i *
50	1.520–6.26 × 10^−3^ *i *
70	1.501–5.60 × 10^−3^ *i *
80	1.443–3.70 × 10^−3^ *i *
90	1.399–2.22 × 10^−3^ *i *
99	1.359–9.16 × 10^−4^ *i *

**Table 2:** Meteorological study of the months of June, July, August, and September 2000, with data on maximum, minimum and average temperatures, relative humidity, wind intensity, and total precipitation registered.

Months	*T*_max⁡_ (°C)	*T*_min⁡_ (°C)	*T*_av._ (°C)	HR (%)	Wind (m/s)	*P*_total_ (mm)
June	31.2	2.2	16.9	62	1.6	8
July	32.4	2.0	17.1	65	1.6	12
August	31.7	2.3	16.7	63	1.4	3
September	30.9	3.2	14.5	71	1.3	26.8

**Table 3:** Number of fires per month and monthly surface burnt according to the type of vegetation affected (trees, nontree woodland, or no-forest land). Total land area affected during the summer of 2000.

Months	No. of Fires	Area Burnt (ha)			
		Trees	Nontree woodland	No-forest land	Total area burnt
June	26	70	389	21	450
July	67	59	612	57	728
August	201	703	8871	275	9848
September	171	922	8075	582	9580

**Table 4:** District where the fire occurred, date of detection and extinction, land area burnt of each type of vegetation (trees, non-tree woodland, and no-forest land) and total land area affected by the large fires (over 500 ha) in the summer of 2000.

Municipality	First detected		Date of extinction		Area burnt (ha)			
	Day	Time (UTC)	Day	Time (UTC)	Trees	Nontree woodland	No-forest mass	Total area burnt
Encinedo (42°16′15′′N, 6°35′39′′W)	18/08/2000	0311	22/08/2000	1930	—	1034	154	1188
Truchas (42°15′40′′N, 6°26′07′′W)	18/08/2000	1650	23/08/2000	0800	179	1539	—	1718
Castrocalbón (42°11′45′′N, 5°58′43′′W)	21/08/2000	1110	22/08/2000	1800	62	773	—	835
Lucillo (42°24′38′′N, 6°18′16′′W)	31/08/2000	1500	03/09/2000	1900	—	1255	—	1255
Castrillo de Cabrera (42°20′25′′N, 6°32′39′′W)	02/09/2000	0600	02/09/2000	1930	—	920	—	920
Villablino (42°55′59′′N, 6°19′00′′W)	06/09/2000	1126	13/09/2000	0700	269	761	—	1,030
Barjas (42°36′40′′N, 6°58′43′′W)	11/09/2000	0830	15/09/2000	1805	33	542	—	575
Bembibre (42°36′54′′N, 6°25′12′′W)	14/09/2000	1605	16/09/2000	1915	104	426	—	530
Villablino (42°55′59′′N, 6°19′00′′W)	17/09/2000	1300	18/09/2000	1900	—	1031	—	1031

**Table 5:** Count median diameter of the fine or accumulation mode (CMD_f_) for each Circulation Weather Type.

CMD_f_ (*μ*m)	Circulation Weather Type
<0.13	ANW-ASW-AW-C-CE-CS-CSE-CSW-E-S-SW
0.13–0.14	A-AN-CW-N-NE-NW-SE-W
0.14–0.15	ANE-CNW
>0.15	AE-CNE

**Table 6:** Weather types and radiative and subsidence thermal inversions during the days with an average number of particles exceeding 2000 particles cm^−3^ (—) means the data are not available.

Day	No. of particles cm^−3^	Circulation weather type	Madrid 0000 UTC		A Coruña 0000 UTC		Santander 0000 UTC	
			Inversions		Inversions		Inversions	
			Radiative (m AGL)	Subsidence (m AGL)	Radiative (m AGL)	Subsidence (m AGL)	Radiative (m AGL)	Subsidence (m AGL)
21/07/00	2084 ± 748	C	208			662	252	
01/08/00	2501 ± 1041	NE	94	494		837		767
07/08/00	2775 ± 3243	E		664		894		
09/08/00	4723 ± 3233	NE	235			462		277
10/08/00	2376 ± 1671	N	361			602	441	634
11/08/00	2380 ± 505	NW	113		—	—	77	798
12/08/00	3755 ± 2562	A				720		
13/08/00	2431 ± 1092	A					102	
14/08/00	3032 ± 1991	A	177		—	—	111	
15/08/00	3014 ± 2090	ANE	—	—				
16/08/00	3101 ± 2911	NE	190			794		
17/08/00	3000 ± 2766	N	132			960		
18/08/00	2240 ± 2304	CNW	161			733	85	
20/08/00	2330 ± 1155	N	65				69	
25/08/00	2157 ± 4291	C	46					324
26/08/00	2760 ± 3202	AN			—	—		
09/09/00	2028 ± 886	E		664	68	606	103	436
16/09/00	2896 ± 2179	C	186					945
22/09/00	2931 ± 2227	ASW	292					111
23/09/00	4912 ± 4005	W	192			295	267	897
24/09/00	5201 ± 3079	A	184	278				
25/09/00	2654 ± 3305	A	268			341	16	
26/09/00	5405 ± 4341	AN	92			968	372	
27/09/00	6972 ± 7035	A	109					
28/09/00	6259 ± 4654	W	—	—	—	—	—	
29/09/00	3625 ± 6597	NW	—	—	—	—	—	
30/09/00	2736 ± 2870	NW						

**Table 7:** Count median diameter of the fine mode (CMD_f_) and relative humidity (RH) for the measurements before the fire, the ones affected by the fire and the ones after the fire.

Measurements prior to those affected by the fire				Measurements affected by the fire				Measurements after those affected by fire			
Day	Time (UTC)	CMD_f_ (*μ*m)	RH (%)	Day	Time (UTC)	CMD_f_ (*μ*m)	RH (%)	Day	Time (UTC)	CMD_f_ (*μ*m)	RH (%)
19/06/2000	1600	0.10	40	20/06/2000	2200	0.18	79	20/06/2000	1600	0.14	43
19/06/2000	1600	0.10	40	20/06/2000	0100	0.20	88	20/06/2000	1600	0.14	43
19/06/2000	1600	0.10	40	20/06/2000	0400	0.16	87	20/06/2000	1600	0.14	43
19/06/2000	1600	0.10	40	20/06/2000	0700	0.16	66	20/06/2000	1600	0.14	43
08/07/2000	1000	0.17	48	08/07/2000	1600	0.11	40	09/07/2000	2200	0.16	89
21/07/2000	1600	0.12	30	22/07/2000	2200	0.19	69	22/07/2000	0400	0.13	83
04/08/2000	0700	0.20	53	04/08/2000	1300	0.11	45	05/08/2000	2200	0.12	74
05/08/2000	1000	0.16	45	05/08/2000	1600	0.10	44	06/08/2000	2200	0.11	86
06/08/2000	1000	0.12	35	06/08/2000	1300	0.10	32	06/08/2000	1900	0.11	67
09/08/2000	0400	0.15	84	09/08/2000	1000	0.11	29	09/08/2000	1600	0.10	38
11/08/2000	0700	0.12	58	11/08/2000	1600	0.15	62	11/08/2000	1900	0.14	86
13/08/2000	2200	0.15	78	13/08/2000	0100	0.15	86	13/08/2000	0400	0.16	90
14/08/2000	0100	0.18	96	14/08/2000	0400	0.15	99	14/08/2000	0700	0.13	67
15/08/2000	1000	0.14	48	15/08/2000	1300	0.12	38	15/08/2000	1600	0.15	42
16/08/2000	1000	0.13	24	16/08/2000	1300	0.08	23	16/08/2000	1600	0.10	24
17/08/2000	0700	0.14	56	17/08/2000	1300	0.10	36	17/08/2000	1900	0.15	71
18/08/2000	1600	0.11	46	18/08/2000	1900	0.10	56	19/08/2000	0100	0.11	82
19/08/2000	0700	0.12	65	19/08/2000	1000	0.10	48	19/08/2000	1900	0.15	42
22/08/2000	1300	0.15	50	22/08/2000	1600	0.15	50	22/08/2000	1900	0.10	62
23/08/2000	1000	0.11	49	23/08/2000	1900	0.12	80	24/08/2000	2200	0.10	82
24/08/2000	1000	0.10	58	24/08/2000	1300	0.10	52	24/08/2000	1600	0.10	44
28/08/2000	1000	0.13	48	28/08/2000	1300	0.10	41	28/08/2000	1900	0.13	69
08/09/2000	1900	0.11	62	09/09/2000	2200	0.11	75	09/09/2000	0400	0.13	80
09/09/2000	0700	0.12	55	09/09/2000	1000	0.12	44	09/09/2000	1600	0.11	48
09/09/2000	1900	0.13	59	10/09/2000	0400	0.11	66	12/09/2000	2200	0.22	97
09/09/2000	1900	0.13	59	10/09/2000	0700	0.12	51	12/09/2000	2200	0.22	97
09/09/2000	1900	0.13	59	10/09/2000	1300	0.10	32	12/09/2000	2200	0.22	97
09/09/2000	1900	0.13	59	10/09/2000	1900	0.11	56	12/09/2000	2200	0.22	97
09/09/2000	1900	0.13	59	11/09/2000	2200	0.13	58	12/09/2000	2200	0.22	97
09/09/2000	1900	0.13	59	11/09/2000	1600	0.12	76	12/09/2000	2200	0.22	97
18/09/2000	1300	0.13	56	18/09/2000	1600	0.12	57	18/09/2000	1900	0.15	70
21/09/2000	1000	0.13	63	21/09/2000	1600	0.10	61	21/09/2000	1900	0.11	92
23/09/2000	1600	0.10	45	24/09/2000	2200	0.10	83	24/09/2000	0700	0.10	71
23/09/2000	1600	0.10	45	24/09/2000	0100	0.13	78	24/09/2000	0700	0.10	71
25/09/2000	0700	0.11	64	25/09/2000	1300	0.10	41	26/09/2000	0700	0.13	62
25/09/2000	0700	0.11	64	25/09/2000	1600	0.08	50	26/09/2000	0700	0.13	62
25/09/2000	0700	0.11	64	25/09/2000	1900	0.11	80	26/09/2000	0700	0.13	62
25/09/2000	0700	0.11	64	26/09/2000	2200	0.14	84	26/09/2000	0700	0.13	62
25/09/2000	0700	0.11	64	26/09/2000	0100	0.10	89	26/09/2000	0700	0.13	62
27/09/2000	0700	0.13	92	27/09/2000	0700	0.12	83	28/09/2000	1000	0.15	98
27/09/2000	0700	0.13	92	27/09/2000	1900	0.10	72	28/09/2000	1000	0.15	98
